# Utilizing epigenetics to study the shared nature of development and biological aging across the lifespan

**DOI:** 10.1038/s41539-024-00239-5

**Published:** 2024-03-21

**Authors:** Laurel Raffington

**Affiliations:** Max Planck Research Group Biosocial—Biology, Social Disparities, and Development, Max Planck Institute for Human Development, Lentzeallee 94, 14195 Berlin, Germany

**Keywords:** Human behaviour, Epigenetics and behaviour

## Abstract

Recently, biological aging has been quantified in DNA-methylation samples of older adults and applied as so-called “methylation profile scores” (MPSs) in separate target samples, including samples of children. This nascent research indicates that (1) biological aging can be quantified early in the life course, decades before the onset of aging-related disease, (2) is affected by common environmental predictors of childhood development, and (3) shows overlap with “developmental processes” (e.g., puberty). Because the MPSs were computed using algorithms developed in adults, these studies indicate a molecular link between childhood environments, development, and adult biological aging. Yet, if MPSs can be used to connect development and aging, previous research has only traveled one way, deriving MPSs developed in adults and applying them to samples of children. Researchers have not yet quantified epigenetic measures that reflect the pace of child development, and tested whether resulting MPSs are associated with physical and psychological aging. In this perspective I posit that combining measures of biological aging with new quantifications of child development has the power to address fundamental questions about life span: How are development and experience in childhood related to biological aging in adulthood? And what *is* aging?

*“We cannot escape our origins, however hard we try, those origins which contain the key - could we find it - to all that we later become.”* – James Baldwin (p. 27, 1955)

## A molecular bridge between aging and development

Humans change dramatically as they age. But what *is* aging? Is it solely characterized by deleterious changes and declining function later in life? Or can it be considered as a process of change that occurs throughout the life course? Similarly, is development solely about growth and improvement in early phases of life? Or is it a continuous process of change that can take place at any stage in the life span?

Traditionally, aging has primarily been studied by gerontologists as a process of change in older adults, while development has predominantly been studied by child developmentalists focused on change in young individuals. However, longitudinal birth cohort studies have revealed relationships between childhood environments and child development with later-life aging. These findings prompt scientific inquiry into understanding how the connection between childhood experiences and outcomes in old age is maintained over so many decades of life^1,2^. These questions about the relationship between development and aging have historically resided in philosophy due to their lack of empirical investigation. Table 1 summarizes such theories of biological aging and their hypothesized relationship between biological aging and development.

**Table 1:** Theories of development and biological aging

Name	Description	References
Antagonistic pleiotropy theory	Aging processes in part are negative biproducts of early life growth. Pleiotropic genes with good early effects would be favored by selection even if these genes had bad effects at later ages. Also known as the “pay later” theory and introduces the idea of a life-history trade-off.	Williams (1957)
Developmental origins of health and disease (DOHaD)	Early life conditions affect later-life health in a manner that is only partially modifiable by later-life experiences. Slow early development is a sign of poor childhood health, so children who develop slower will age faster in midlife, with shorter longevity.	Gillman (2005); Barker (2007); Barker (1990); Hayward & Gorman (2004)
Developmental theory	Aging and development are coupled and regulated by the same mechanisms.	Zwaan (2003); deMagalhaes, Church. (2005)
Disposable soma theory of aging	Competition for metabolic resources between processes such as growth, reproduction, and cellular maintenance lie at the heart of the aging process. Human aging is seen as a gradual and interrelated loss of integrity in every organ system due to a loss of somatic maintenance and repair.	Kirkwood (1977, 2000)
Early life programming / sensitive periods	During early ontogeny (e.g., prenatally and early postnatal life), an individual’s brain and body are modified to maximize survival and reproduction in their predicted future environment. Early life environments may “program” biological parameters for accelerated aging and disease risk, even if clinical signs of age-related disease may not be evident until decades later.	Ellis & Del Diudice (2019); Belsky (2019)
Evolutionary life history theory	Age-acceleration mechanisms are lasting, so children who develop faster also age faster in midlife, with shorter longevity.	Hill & Kaplan (1999); Kaplan (2000).
Ground Zero Model of Organismal Life and Aging	Biological aging represents an integrative measure of deleterious changes that occur during organismal life. Biological aging commences prenatally in the mid-embryonic state, but is distinct from development, which is a genetic program that begins at conception and ends roughly at age 20 with the aim to build a fit organism.	Gladyshev (2021)
Lifespan psychology	Development and aging are often used synonymously. In some conceptualizations, development and aging describe largely shared processes and constitute “two sides of the same coin”. In other descriptions, processes of maturation and aging are thought to be evolutionarily distinct, but result in mechanisms that occur throughout life span development. Development primarily describes age-related change in adaptive capacity (i.e. plasticity). With increasing age, losses are on an increasing trajectory and gains are on a decreasing trajectory, but gains and losses coexist across the entire life span. This conception of development differs from biological maturational models, which posit universal, cumulative-integrative, and sequential movement toward higher levels of functioning.	Baltes, Staudinger, & Lindenberger (1999); Belsky (2019); Etzel, Garrett‐Petters, & Shalev (2023)
Mutation accumulation theory	Later-life aging occurs as the result of an “evolutionary selection shadow”. Wild animals do not live long enough to grow old. Therefore, natural selection has limited opportunity to exert a direct influence on senescence. This selection shadow allows a wide range of alleles with late deleterious effects to accumulate over generations.	Medawar (1946)
Plasticity	Any developmental outcome is but one of numerous possible outcomes. Plasticity is triggered when experiential forces interact with genetic programs in the maturation of species-common functions, but is also involved in forms of learning that make individuals unique. The notion of plasticity can be taken so far as to challenge the concept of any genetically-determined outcome. Older adults continue to possess sizeable plasticity, but there are robust aging-related losses in plasticity.	Lindenberger & Lövden (2019)
Pseudo-programmatic theory of aging	Aging is evolutionarily conserved and intertwined with developmental processes across all mammals. The process of aging is a consequence of the process of development, and the ticking of epigenetic clocks reflects the continuation of developmental processes. Epigenetic clocks provide a continuous readout of age from early development to old age in all mammals, as this feature underlies the continuous and largely deterministic process of aging from conception to tissue homeostasis.	Lu et al. (2023); Magalhães (2012)

The table reports selected theories of development and biological aging in alphabetical order, including the name, description in regards to the relationship of development and aging, and references.

In this perspective I argue that new measurement technologies now provide an opportunity to empirically explore the relationship between aging and development. Specifically, biological aging has been quantified in DNA-methylation (DNAm) samples of older adults and applied as so-called “methylation profile scores” (MPSs) in separate target samples, including samples of children. These studies suggest that (1) biological aging can be quantified early in the life course, decades before the onset of aging-related diseases, (2) is affected by common environmental predictors of childhood development, and (3) shows overlap with developmental processes, such as pubertal timing. Because the MPSs were computed using algorithms developed in adults, these findings indicate a molecular link between childhood environments, development, and adult biological aging.

**Fig. 1:**
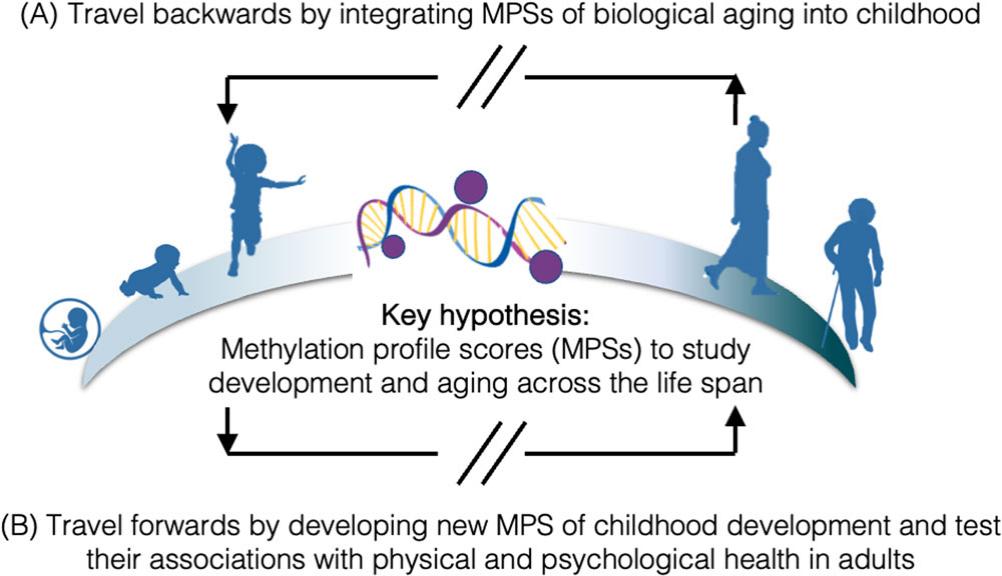
Applying methylation profile scores (MPSs) to study development and aging across the life span. **A** Recent research has traveled backwards in time by integrating MPSs of biological aging developed in adults into cohorts of children and adolescents. **B** Researchers have not yet traveled forwards by developing new MPS of childhood development and tested their associations with physical and psychological health in adults.

Yet, if DNAm measures can be used as a molecular bridge to connect development and aging, previous research has only traveled one way, by applying MPSs to child samples that were developed in adults (**pathway A in** Fig. 1). Researchers have not yet attempted to travel forward across the life span, by developing algorithms that reflect child and adolescent development, and testing whether resulting methylation profiles are associated with physical and psychological aging in adults (**pathway B in** Fig. 1). By integrating assessments of biological aging with novel quantifications of childhood development, we gain the ability to explore fundamental questions about the human lifespan: What is the connection between childhood experiences and development, and the process of biological aging in adulthood? And what, in essence, is aging?

This article comprises three sections. First, I briefly introduce recent DNAm measures of biological aging and related phenotypes. I discuss a major methodological challenge in this field that is commonly referred to as the “tissue issue”, which is an especially salient problem for studies comparing lifespan cohorts. Second, I highlight nascent epigenetic studies that have quantified biological aging early in the life span, and in association with a range of environmental, physiological, and psychological phenotypes. Third, I propose future directions that leverage epigenetic measures to quantify child development across the lifespan.

## Methylation profile scores of biological aging and related phenotypes

Epigenetic mechanisms, including DNAm, regulate the expression of genes and are involved in the processes that embed early life privilege and disadvantage into our biology^3^. DNAm is the reversible addition of a methyl group onto the fifth carbon of a cytosine residue in DNA. DNAm is usually a stable, genetically-influenced epigenetic mark that underpins the lifelong maintenance of cellular identity, but it can also be a dynamic developmental process that changes with age and environmental inputs^4^. Technological advances now allow for the measurement of genome-wide DNAm in thousands of individuals. These studies typically test the associations between individual DNAm probes and exposures, phenotypes or diseases.

The results from such discovery studies can be used to generate “methylation profile scores” (MPSs) in independent data sets, including samples of children, which can then be examined in relation to a wide range of measured variables. Table 2 summarizes MPSs of biological aging and related phenotypes. The first generation of MPSs were trained on chronological age and are commonly called “epigenetic clocks”^5^. These studies showed that MPS can be developed to very closely track chronological age with correlation coefficients upwards of *r* = 0.95, even when DNAm is quantified across multiple tissues. Therefore, DNAm is now considered a “hallmark of aging”^6^. (For computational descriptions of MPSs compared to polygenic scores see^7^ and for comprehensive reviews on multiple types of aging biomarkers see^8,9^.)

**Table 2:** Methylation profile scores

Methylation profile score	Tissue	Training criterion	Interpretation
*Chronological age*			
(Horvath, 2013)	Multiple	Adults across 82 different datasets across entire lifespan	Age predicted by DNA methylation
(Hannum et al., 2013)	Blood	Adult volunteers at UC San Diego, University of Southern California, and West China Hospital aged 19-101 years	Age predicted by DNA methylation
*Cross-sectional physiology*			
PhenoAge (Levine et al., 2018)	Blood	Adults from the InCHIANTI Study aged 21-100 years	Age at which average mortality risk in NHANES III matches the mortality risk predicted by the PhenoAge algorithm
GrimAge (Lu et al., 2019)	Blood	Adults from the Framingham Heart Study Offspring cohort aged 53-73 years	Age at which average mortality risk in the Framingham Heart Study Offspring cohort matches predicted mortality risk
DNAm of inflammation (Ligthart et al., 2016)	Blood	Serum hs-CRP in adults from multiple cohorts with mean ages 60-87 years controlling for age, sex, and BMI	Higher score indicates a DNAm profile that more closely resembles the DNAm profile of adults with higher hs-CRP.
Body mass index (BMI) (Wahl et al., 2017)	Blood	Body mass index in adults from multiple cohort ages 51 to 70 controlling for age and sex.	Higher score indicates a DNAm profile that more closely resembles the DNAm profile of adults from multiple cohorts and ancestries with higher BMI
*Longitudinal physiology*			
DunedinPACE-pace of aging (D. W. Belsky et al., 2022)	Blood	Change over 19-years of follow-up in 19 system-integrity biomarkers, including hs-CRP and BMI (repeated at ages 26, 32, 38, and 45 years).	Years of physiological decline experienced per 1 y of calender time. Values > 1 indicate accelerated aging. Values < 1 indicate slowed aging
*Cognitive health*			
Epigenetic-*g (McCartney* et al.*, 2022)*	Blood	General cognitive functioning in adults ages 18 to 93 controlling for age, sex, and BMI.	Higher score indicates a DNAm profile that more closely resembles the DNAm profile of adults from the Generation Scotland Study who performed higher on tests of general cognitive function, including logical memory, digit symbol test score, verbal fluency and vocabulary.

The table reports selected methylation profile scores applied in studies referenced in the present article. For each measure, the table lists the tissue used to extract DNA-methylation in the discovery sample, training criterion used to develop the measure, and the interpretation of the measure’s values.

Second generations of DNAm measures of biological aging were created using cross-sectional measures of multisystem physiological functioning, health, and mortality (e.g., GrimAge, PhenoAge^10,11^). Third generations of DNAm measures of biological aging were created using longitudinal measures of multisystem physiological decline within the same people (e.g., DunedinPACE-pace of aging^12^).

These next-generation DNA-methylation measures of biological aging developed to predict mortality risk and physiological decline are more predictive of morbidity and mortality than the original epigenetic clocks trained on chronological age^13,14^. These new measures also show consistent evidence of more advanced and faster biological aging in adults exposed to socioeconomic disadvantage, race and ethnic marginalization, and, in adults, these differences in biological aging partially account for socioeconomic and racial health disparities^14–17^.

A related set of studies has developed MPS of inflammation^18^, body mass index (BMI^19,20^), and cognitive performance on the basis of adult blood samples (epigenetic-*g*^21^; Table 2). Similar to measures of biological aging, these MPS have been found to predict health, mortality and cognitive performance in separate adult target samples.

### The tissue issue

Emerging epigenetic studies have quantified biological aging in DNAm early in the life course. In adults, DNAm measures of biological aging are typically developed using venous blood, which is considered the gold-standard sampling method. In children, DNA is commonly collected from saliva, cheek, and dried-blood spot DNA, as it is less invasive, can be sampled via postal kits, and often has higher participation rates than blood (saliva 72% vs. blood 31%^22^). Because DNAm patterns encode cell identity, they differ by tissue. Therefore, it is important to consider the cross-tissue correspondence of MPSs created based on different DNA sampling methods.

Previous findings provide evidence for good saliva-blood cross-tissue correspondence for examined MPSs. First, samples are partially composed of the same cell types: blood samples consist of 100% immune cells, saliva in children consist of approximately ~65% immune cells and ~35% epithelial cells^23^. While statistical corrections for people’s cell composition are common, immune cell DNAm appears to be particularly sensitive to early life exposures and aging-related inflammatory processes (called “inflammaging”) that can affect multiple tissues, including neurons^9,24^. Second, the MPS of the pace of biological aging computed in both blood and saliva tissues from the same persons show high cross-tissue rank-order stability^25^. Third, children’s saliva MPS of BMI computed on the basis of an adult blood study reflects monozygotic twin differences in child body size – one of the most stringent tests of biomarker sensitivity^26^. Moreover, this saliva MPS of BMI indicates bidirectional longitudinal associations with BMI across adolescence, incrementally predicting children’s future BMI^26^.

While the saliva-blood cross-tissue correspondence of these MPSs may be acceptable, the cross-tissue correspondence for those same MPS appears to be lower for cheek samples (i.e., buccal tissue^27^). This may be because buccal cells consist of only ~20% immune cells and ~80% epithelial cells^28,29^. Lower rates of immune cell DNAm may weaken signals of early life adversity and aging-related inflammatory processes.

The cross-tissue correspondence of MPSs remains a major methodological concern for future research. Both scientific research and commercial use appear to be moving away from collecting venous blood to more accessible tissues. Thus, a variety of tissues in a variety of age groups will be used to derive MPSs in the near future. Given the increased availability of different MPSs, it can already be challenging to decide which MPSs to employ. To further complicate this tissue issue, it is likely that cross-tissue correspondence will differ by MPS, depending on the tissue(s) and phenotype(s) they were developed to predict and how that phenotype is reflected across tissues, which may depend on ontogenetic processes. Studies that collect multiple tissue types from the same people of differing ages will be an expensive but essential step to addressing tissue-related measurement invariance of MPSs.

Keeping these limitations in mind, in the next section I will discuss recent empirical evidence, largely derived from saliva MPSs, that (1) biological aging can be quantified early in the life course, (2) is affected by common environmental predictors of childhood development, and (3) reveals overlap with developmental processes.

## Quantifying biological aging early in the life span

The vast majority of studies of human aging examine older adults. Yet, it has long been observed that age-related diseases and mortality could be predicted from early life development, including intrauterine growth retardation, low self-control in childhood, and early life adversity^30–32^. Accordingly, there has recently been a paradigm shift in the gerosciences that anchors the onset of biological aging to the prenatal period as opposed to later in the life course after the presumed completion of development and onset of reproductive age^33^. Nevertheless, to-date little is known about biological aging in young humans^2^.

Recent epigenetic studies have attempted to quantify biological aging in DNAm of children, adolescents, and young adults. In our own research, we computed MPSs – originally developed in adults—in over *n* = 3000 children and adolescents from two sociodemographically diverse US cohorts that combine twin and longitudinal study designs, the Texas Twins Project and the Future Families and Child Well-Being Study. First, we found that these MPSs when applied to children showed means, distributions, and associations with chronological age that were in line with MPS applications in adults: Older children had MPSs of older chronological age, higher inflammation, higher BMI, higher cognitive performance, and a faster pace of aging^25,26,34^.

Second, twin analyses suggested that MPSs capture genetic, systematic and stochastic environmental sources of variation^26,34^. Third, analyses of longitudinal repeated measures of MPSs found that MPSs are stable across childhood and adolescence, though less stable than reports in adults^26^(deSteiguer et al., in press; Koss et al., in press). This demonstrates that a substantial amount of between-person variation in MPSs arises prior to late childhood. Our and other studies suggest that biological aging can be quantified long before the onset of aging-related diseases, potentially even shortly after birth^35,36^. There is a notable lack of studies examining the longitudinal stability and change of MPSs in early childhood, because repeated measurements of DNA methylation in young children are still rare and the idea to examine biological aging in children is relatively new. Similarly, very few studies have explored the clinical relevance of MPSs in pediatric cohorts, though there is some evidence to suggest that they may, for example, help identify survivors of childhood cancer at increased risk for early-onset obesity, morbidity, and mortality^37^.

### Biological aging is affected by common environmental predictors of childhood development

Individuals who are socioeconomically disadvantaged or marginalized based on their racial and ethnic identity tend to develop aging-related diseases at younger ages and experience earlier mortality as compared to individuals who are wealthier and White^38,39^. One mechanism hypothesized to link social determinants of health with shorter healthy lifespan is an acceleration of biological aging^40,41^. Studies in adults have found that next-generation DNA-methylation measures of biological aging developed to predict mortality risk and physiological decline show consistent evidence of more advanced and faster biological aging in adults exposed to socioeconomic disadvantage and racial/ethnic marginalization^14^. Different manifestations of racism, including institutional, environmental, and interpersonal dynamics of racialization, may contribute to higher average levels of chronic stress, inflammation, and accelerated multi-system biological aging among marginalized communities^42–45^.

Given previous research that age-related diseases and mortality can be predicted from early life factors, we probed whether MPSs of biological aging and related phenotypes were sensitive to childhood socioeconomic and racial/ethnic marginalization measured in real-time. In the Texas Twin Project, we found children from underserved families to have MPSs indicating advanced biological age, a faster pace of aging, higher chronic inflammation, higher BMI, and lower cognitive health^26,34,46^. Some of these findings have been replicated in buccal samples from the German SOEP-G sample^27^, as well as the Future Families and Child Well-Being Study, where we found socioeconomic contexts at birth relative to concurrent socioeconomic contexts in childhood and adolescence to be most strongly associated with MPS of BMI in childhood and adolescence (^26,47^; for review see also^48^). Racial and ethnic disparities in children’s biological aging were reduced, but remained visible, after statistically accounting for perinatal and postnatal covariates, including the substantially higher risk of socioeconomic disadvantage in racially marginalized communities. This suggests that exposure to discriminatory policies and actions, especially in low-income areas, contribute to the emergence of racial disparities in physiological burden in the first decades of life^49^.

Because the MPSs were computed using algorithms developed in adults, these studies indicate a molecular link between childhood environments and adult biological aging^25^. They are consistent with the hypothesis that childhood socioeconomic and racial/ethnic marginalization affects not only the outcome of accelerated biological age, but also the *pace* of aging across the life span. More rapid biological aging in underserved environmental contexts beginning early in ontogeny may constitute the start of trajectories toward earlier onset of adult disease.

An important implication of this new knowledge is that future interventions will need to be scheduled early in life if they hope to prevent accelerated aging, age-related disease, and improve the quality of longer lives^2,50^. Measurement of MPSs in childhood affords a unique opportunity to provide insight regarding the ways in which early experiences may set parameters for physical and psychological health even though clinical signs of disease may not be evident until decades later. Future research with repeated DNA-methylation measures from birth can explore how early in life these associations first become apparent.

Early ontogenetic development is especially sensitive to environmental contexts, given the rapid pace of development and high developmental plasticity. Developmental theories posit that epigenetic mechanisms during the prenatal and early‐life periods refine the genetic program to be optimally responsive to present and future environmental challenges^51^. Experiences during this period appear to exert lasting effects on MPSs of biological aging quantified in older adults^52^. It remains to be seen whether MPSs are sensitive to experimental manipulations in socioeconomic resources in real-time, such as cash gifts in early childhood^53^.

### Biological aging shows overlap with developmental processes

Amongst children, early life adversity has been observed to accelerate both biological aging and developmental processes, such as earlier pubertal development, earlier sexual functioning, and a faster pace of brain development^54–56^. Early in life, accelerated aging has been theorized to be adaptive in the sense that achieving milestones of growth and sexual maturity on an accelerated timeline within a threatening or impoverished environment can increase the likelihood of reproduction before an untimely death^57^. Thus, within an evolutionary framework aging and reproductive development are intrinsically linked^58^.

We quantified MPSs of biological aging in children and adolescents to examine whether accelerated biological age and a more rapid pace of aging bears any relation to pubertal development. In 8-18-year-olds from the Texas Twin Project, we found no associations between pubertal timing and accelerated biological age^25^. We did, however, find a somewhat faster pace of aging in girls who had experienced their first menses compared with same-aged girls who had not. We also found that children who were more advanced in their pubertal development had MPSs indicating higher levels of chronic inflammation, even after accounting for socioeconomic disadvantage^34^. Other cohort studies have found earlier pubertal timing to be associated with accelerated biological age in 11-to-13-year-old Finish adolescents^36^ and in premenopausal women^59^.

Since girls begin and complete pubertal development earlier than boys^60,61^, sex differences in MPSs of biological aging can further inform associations with pubertal maturation. In contrast to studies in adults that consistently find more advanced biological age in males^62^, we found that adolescent girls had a faster pace of aging compared to boys^34^. Collectively, these studies suggest that earlier and more rapid pubertal development is weakly associated with accelerated biological age and a faster pace of biological aging, potentially more so in girls than boys. This is consistent with the hypothesis that individuals who reproductively develop faster in adolescence also decline somewhat faster later in life. It may indicate that reproductive development early in life shares some biological mechanisms with processes of decline later in life.

Beyond pubertal development, we have found MPSs of the pace of aging, chronic inflammation, and cognitive performance in adults to be associated with children’s psychological development, including lower perceptual and verbal reasoning and higher parent-reported internalizing symptoms in children from the Texas Twin Project^34,63^. Notably, we found that a MPS of cognitive performance in adults (i.e., Epigenetic-g) explains 11% of the variance in children’s in-laboratory math performance^34^. Our findings, and those of other researchers^35,64^, suggest that MPS quantifications of biological aging and related phenotypes show overlap with both physical and psychological development, and can be used to study the childhood roots of health and well-being.

## Quantifying development across the life span

If epigenetic measures can be used to connect development and aging, previous research has only traveled one way, by applying MPSs to child samples that were developed in adults (**pathway A in** Fig. 1). These studies can situate aging processes at early and middle life stages to better understand trajectories of aging across the life course. Researchers have not yet quantified epigenetic measures that reflect fetal or child development, and tested whether resulting MPSs are associated with physical and psychological aging in adults (**pathway B in** Fig. 1). While several MPSs have been developed to estimate gestational age or chronological age using infant’s or children’s DNAm^8,65,66^, no studies have examined the relationship between gestational age and biological aging MPSs later in life. Moreover, similar to first-generation MPSs trained on chronological age in adults, we have found that these MPSs of age in children are not consistently associated with social determinants of health or psychological phenotypes compared to next-generation MPSs of physiological decline in adults^25^.

Critically, researchers have not yet quantified epigenetic measures that reflect childhood developmental phenotypes and tested whether resulting MPSs are associated with physical and psychological aging in adults. For instance, would a MPS developed in adolescents to quantify pubertal timing predict onset of menopause (an indicator of reproductive aging), health, and mortality in adults? Would a MPS developed in young children to quantify the multi-system pace of development—as indicated by measures of body mass index, inflammatory markers, and cardiovascular health – be correlated with accelerated biological age or a faster pace of biological aging?

Molecular quantifications of development would allow researchers to test competing hypotheses as to whether fast versus slow development early in life forecast faster aging in midlife (Table 1). One hypothesis, from evolutionary life history theory, says age-acceleration mechanisms are lasting, so children who develop faster also age faster in midlife, with shorter longevity (T. Moffitt, personal communication, July 15, 2023; Hill & Kaplan 1999; Kaplan 2000). An opposing hypothesis from the developmental origins of health and disease theory says slow early development is a sign of poor childhood health, so children who develop slower will age faster in midlife, with shorter longevity (Gillman 2005). If both theories are true, we would expect MPSs of development to show a non-linear relationship with MPSs of biological aging, indicating that both fast and slow early development is related to faster aging in midlife (T. Moffitt, personal communication, July 15, 2023). Moreover, these epigenetic studies can clarify whether developmental delay and acceleration are governed by similar biological pathways, which may differ by domain.

**Fig. 2:**
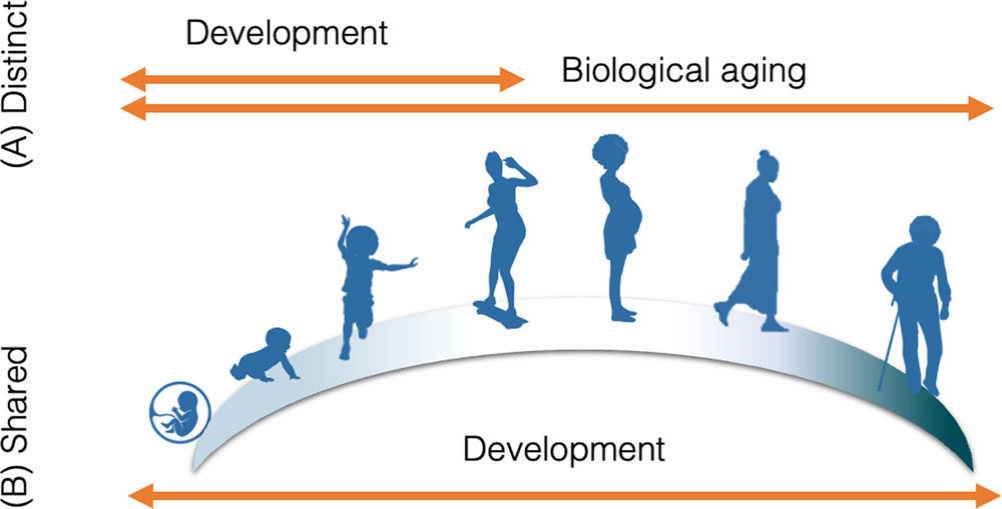
Theories differ in their hypothesized relationship of development and aging. **A** In many biological models, the purpose and mechanism of development and aging are distinct. While biological aging may onset early in the life course, development is restricted to the first decades of life. **B** In contrast, life span psychology often uses development and aging synonymously. In some conceptualizations, development and aging are largely shared and constitute “two sides of the same coin”. In other theories, processes of maturation and senescence are thought to be evolutionarily distinct, but result in developmental mechanisms that co-occur throughout the life span.

In contrast, the Ground Zero Model of Organismal Life posits that development is distinct from biological aging and ends roughly at age 20 (Gladyshev, 2020; see Fig. 2). Thus, we may expect MPSs of development to be unassociated with MPSs of biological aging. Furthermore, given that aging is thought to be under less genetic control than development with more stochastic variation that accrues over the life course, we may purport that MPSs of aging developed in older adults should be more variable and less heritable than MPSs of development. Thus, combining measures of biological aging with quantifications of child development can provide a new avenue of research on how experience and development in childhood are related to biological aging.

Moreover, MPSs of child development could help address questions of whether children may benefit from being “developmentally” older or younger relative to children of the same chronological age in terms of health, educational, and social outcomes. Nascent neuroscientific studies suggest that faster pace of brain development very early in life that then slows down in mid-childhood may be associated with higher cognitive performance^56^. Yet, the use of different brain metrics at different scales makes it very difficult to identify an overarching pattern^56^. Distilling multi-system developmental processes into a single MPS per children could substantially help contextualize these findings.

To develop the most powerful MPSs of child development, researchers would preferably derive data from prospective birth cohorts across multiple developmental systems (e.g., physiological, motoric, cognitive), measured at repeated time points starting at birth or earlier. While sample sizes for MPSs computation are typically several orders of magnitude smaller than those needed for DNA-based summary scores (i.e., thousands rather than millions), larger and diverse discovery samples are likely to yield more powerful and generalizable biomarkers (i.e., several thousands of individuals of diverse socioeconomic, racial, and geographic background). Cohorts including children with more severe degrees of developmental delay and acceleration could potentially also improve MPSs performance and clinical utility.

In line with MPS quantifications of the pace of biological aging in adults, studies aiming to develop new MPSs of child development could first generate latent factors of within-person change across physiological systems early in life and then use elastic net regression models to train DNAm on this latent factor^12,67^. The resulting algorithm can then be applied to derive a MPS of the pace of child development in separate target samples of various ages. Repeated measures can also be used to disentangle markers that contribute to stability, but also to changes in processes of development across time.

These types of datasets are, of course, immensely expensive, effortful, and slow to generate. Yet, as the availability of DNAm data from existing cohort studies increases, researchers will soon be able to increase their statistical power by meta-analyzing across studies (cf. Pregnancy and Childhood Epigenetics consortium). For new data collection efforts, DNAm sampling from multiple tissues can help address tissue-related measurement invariance of derived MPSs. Collection of other aging biomarkers, including mitochondrial DNA and RNA, should be considered as these may soon be implemented in multi-omics approaches to quantify aging. Moreover, molecular quantifications of development may identify novel candidate biological pathways that may not be visible in adult samples, after accumulation over many years; potentially yielding easily implementable biomarkers relevant to preventive and therapeutic targets from childhood into old age.

## Conclusion

Novel measures quantified in DNAm suggest that aging is not a phenomenon confined to the old. These new tools have potential to help evaluate how early life factors come to predict later life health and well-being. This is of relevance to understanding development of disparities in health and mortality, but also to psychological traits, like mental health and cognition, as indicated by emerging psychological studies. Ultimately, these measures may highlight effective interventions and preventative measures, decades before effects on aging-related chronic disease or mortality would be apparent. Combining measures of biological aging with new quantifications of child development has the power to address a fundamental question about life span: how are experience and development in childhood related to biological aging?

### Reporting summary

Further information on research design is available in the Nature Research Reporting Summary linked to this article.

### Supplementary information

Reporting summary
